# Supplementary material for: Risk of Adverse Drug Events Following the Virtual Addition of COVID-19 Repurposed Drugs to Drug Regimens of Frail Older Adults with Polypharmacy
Source: J Clin Med. 2020 Aug 10;9(8):2591. doi: 10.3390/jcm9082591 (PMC7463624; doi:10.3390/jcm9082591)
Supplement: Supplementary file 1 [file jcm-09-02591-s001.pdf]

**Table S1.** The top 25 prescribed medications.

| Medication Name | Number of patients (N) | Prevalence (%) |
|---|---|---|
| Cholecalciferol | 6,776 | 54% |
| ASA | 5,283 | 42% |
| Atorvastatin | 4,135 | 33% |
| Acetaminophen | 3,507 | 28% |
| Amlodipine | 3,231 | 26% |
| Docusate sodium | 3,217 | 25% |
| Metoprolol | 3,062 | 24% |
| Lisinopril | 2,847 | 22% |
| Gabapentin | 2,810 | 22% |
| Thyroxine | 2,642 | 21% |
| Furosemide | 2,629 | 21% |
| Pantoprazole | 2,402 | 19% |
| Sennosides | 2,396 | 19% |
| Metformin | 2,263 | 18% |
| Vitamin B12 | 1,811 | 14% |
| Fluticasone | 1,727 | 13% |
| Losartan | 1,726 | 13% |
| Calcium carbonate | 1,709 | 13% |
| Melatonin | 1,591 | 12% |
| Sertraline | 1,583 | 12% |
| Potassium chloride | 1,521 | 12% |
| Trazodone | 1,479 | 11% |
| Famotidine | 1,422 | 11% |
| Omeprazole | 1,395 | 11% |
| Hydrochlorothiazide | 1,359 | 10% |

**Table S2.** The most common diseases.

| Disease name category | ICD10 code | Number of patients (N) | Prevalence (%) |
|---|---|---|---|
| Essential hypertension | I10 | 4567 | 36% |
| Hyperlipidemia | E78 | 4200 | 34% |
| Pain<br>(acute pain due to trauma; other chronic pain; chronic pain syndrome; unspecified; pain hip or knee) | G89, R52, M25 | 4178 | 33% |
| Vitamin D deficiency | E55 | 3778 | 30% |
| Constipation<br>(slow transit constipation; drug induced constipation) | K59 | 3759 | 30% |
| Type 2 diabetes mellitus<br>(T2D; T2D with diabetic nephropathy; T2D with hyperglycemia; T2D with other specified complication; T2D with unspecified complications) | E11 | 3549 | 28% |
| Gastro-esophageal reflux disease (GERD) | K21 | 2942 | 23% |
| Depression<br>(major depressive disorder, recurrent, moderate; recurrent severe without psychotic features; recurrent, in full remission; single episode, moderate; single episode, severe with psychotic features; single episode, in partial remission; single episode unspecified) | F33, F32 | 2405 | 19% |
| Rhinitis<br>(vasomotor rhinitis; other seasonal allergic rhinitis) | J30 | 2036 | 16% |
| Chronic obstructive pulmonary disease (COPD)<br>(with exacerbation; unspecified) | J44 | 1800 | 14% |
| Insomnia | G47 | 1587 | 12% |
| Hypothyroidism | E03 | 1521 | 12% |
| Anxiety<br>(panic disorder; episodic paroxysmal anxiety; generalized anxiety disorder) | F41 | 1365 | 11% |
| Dry eye syndrome | H04 | 1361 | 10% |
| Coronary heart disease<br>(atherosclerotic heart disease of coronary artery without angina pectoris; ischemic cardiomyopathy; atherosclerosis of coronary artery bypass graft(s) without angina pectoris; chronic total occlusion of coronary artery; chronic ischemic heart disease, unspecified) | I25 | 1328 | 10% |
| Heart failure<br>(left ventricular failure, unspecified; chronic systolic congestive heart failure; combined systolic congestive and diastolic congestive heart failure) | I50 | 1141 | 9% |
| Radiculopathy<br>(site unspecified; thoracic region; low back pain; other dorsalgia) | M54 | 1001 | 8% |
| Atrial fibrillation | I48 | 986 | 8% |
| Glaucoma | H40 | 982 | 8% |
| Osteoporosis<br>(age-related without current pathological fracture) | M81 | 912 | 7% |
| Asthma | J45 | 785 | 6% |
| Benign prostatic hyperplasia | N40 | 767 | 6% |

**Table S3.** Concomitant CYP2C8 interacting drugs including CYP2C8 inhibitors, CYP2C8 inducers and potential interacting CYP2C8 competitive substrates with HCQ or CQ.

| CYP2C8 drug names | Number of patients, N (%) |
|---|---|
| Ibuprofen | 224 (1.8%) |
| Loperamide | 154 (1.2%) |
| Trimethoprim | 138 (1.1%) |
| Pioglitazone | 109 (0.9%) |
| Primidone | 102 (0.8%) |
| Repaglinide | 43 (0.4%) |
| Phenobarbital | 35 (0.3%) |
| Gemfibrozil | 32 (0.3%) |
| Teriflunomide | 5 (0.3%) |
| Zafirlukast | 5 (0.04%) |
| Abiraterone | 3 (0.02%) |
| Rifampicin | 1 (0.01%) |

**Table S4.** The medication risk factor scores for each factor for the population under study at baseline and following the addition of COVID-19 repurposed drugs.

| Drug Name | Mean (SD) | Median | *p*-values * |
|---|---|---|---|
| FDA Adverse Event Reporting System (FAERS) score[t] | | | |
| Baseline | 3.3 (0.9) | 3 | |
| HCQ | 3.5 (0.8) | 4 | <0.0001 |
| HCQ + AZ | 3.6 (0.7) | 4 | <0.0001 |
| CQ | 3.5 (0.8) | 4 | <0.0001 |
| CQ + AZ | 3.7 (0.7) | 4 | <0.0001 |
| LPV/r | 3.7 (0.7) | 4 | <0.0001 |
| CYP450 drug interaction burden score | | | |
| Baseline | 4.5 (3.2) | 5 | |
| HCQ | 6.1 (2.9) | 6 | <0.0001 |
| HCQ + AZ | 6.1 (2.9) | 6 | <0.0001 |
| CQ | 5.5 (3.2) | 5 | <0.0001 |
| CQ + AZ | 5.5 (3.2) | 5 | <0.0001 |
| LPV/r | 9.6 (3.2) | 10 | <0.0001 |
| Anticholinergic burden score | | | |
| Baseline | 2.0 (1.8) | 1 | |
| HCQ | 2.0 (1.8) | 1 | 0.97 |
| HCQ + AZ | 2.0 (1.8) | 1 | 0.97 |
| CQ | 2.0 (1.8) | 1 | 0.97 |
| CQ + AZ | 2.0 (1.8) | 1 | 0.97 |
| LPV/r | 2.0 (1.8) | 1 | 0.97 |
| Sedative burden score | | | |
| Baseline | 2.8 (1.3) | 3 | |
| HCQ | 2.8 (1.3) | 3 | 0.94 |
| HCQ + AZ | 2.8 (1.3) | 3 | 0.94 |
| CQ | 2.8 (1.3) | 3 | 0.94 |
| CQ + AZ | 2.8 (1.3) | 3 | 0.94 |
| LPV/r | 2.8 (1.3) | 3 | 0.94 |
| LQTS score | | | |

| | | | |
|---|---|---|---|
| Baseline | 1.7 (2.5) | 0 | |
| HCQ | 2.8 (3.0) | 2 | <0.0001 |
| HCQ + AZ | 4.4 (3.0) | 7 | <0.0001 |
| CQ | 2.8 (3.0) | 2 | <0.0001 |
| CQ + AZ | 4.4 (2.9) | 7 | <0.0001 |
| LPV/r | 3.0 (3.0) | 2 | <0.0001 |
| Fexofenadine | 1.7 (2.5) | 0 | 1.0 |
| Terfenadine | 3.1 (3.0) | 2 | <0.0001 |
| Terfenadine + AZ | 4.5 (2.9) | 7 | <0.0001 |

* *p*-values. Wilcoxon signed-rank test analysis was used to compare composite individual risk score before and after addition of repurposed drugs for each participant. ŧ The FAERS score uses the FDA Adverse Event Reporting System to compute risk of adverse drug events (ADE) which is based on computation of a drug regimen relative odds ratio for ADE using the FDA pharmacovigilance database. AZ, azithromycin; CQ, chloroquine; HCQ, hydroxychloroquine; LPV/r, lopinavir boosted with ritonavir; MRS™, medication risk score; SD, standard deviation.
